# Duration of Untreated Psychosis Is Associated with Temporal and Occipitotemporal Gray Matter Volume Decrease in Treatment Naïve Schizophrenia

**DOI:** 10.1371/journal.pone.0083679

**Published:** 2013-12-31

**Authors:** Xiaofeng Guo, Jun Li, Qinling Wei, Xiaoduo Fan, David N. Kennedy, Yidong Shen, Huafu Chen, Jingping Zhao

**Affiliations:** 1 Institute of Mental Health, the Second Xiangya Hospital, Central South University, Changsha, China; 2 Key Laboratory for NeuroInformation of the Ministry of Education, School of Life Science and Technology, University of Electronic Science and Technology of China, Chengdu, China; 3 Department of Psychiatry, 3rd Affiliated Hospital of Sun Yat-sen University, Guangzhou, China; 4 UMass Memorial Medical Center, University of Massachusetts Medical School, Worcester, Massachusetts, United States of America; Institute of Automation, Chinese Academy of Sciences, China

## Abstract

**Background:**

Long duration of untreated psychosis (DUP) is associated with poor treatment outcome. Whether or not DUP is related to brain gray matter volume abnormalities in antipsychotic medication treatment naïve schizophrenia remains unclear at this time.

**Methods:**

Patients with treatment-naïve schizophrenia and healthy controls went through brain scan using high resolution Magnetic Resonance Imaging. DUP was evaluated using the Nottingham Onset Schedule (NOS), and dichotomized as short DUP (≤ 26 weeks) or long DUP (>26 weeks). Voxel-based methods were used for volumetric measure in the brain.

**Results:**

Fifty-seven patients (27 short DUP and 30 long DUP) and 30 healthy controls were included in the analysis. There were significant gray matter volumetric differences among the 3 groups in bilateral parahippocampus gyri, right superior temporal gyrus, left fusiform gyrus, left middle temporal gyrus, and right superior frontal gyrus (p's<0.01). Compared with healthy controls, the long DUP group had significantly smaller volume in all these regions (p's <0.05). Compared with the short-DUP group, the long-DUP group had significantly smaller volume in right superior temporal gyrus, left fusiform gyrus, and left middle temporal gyrus (p's<0.01).

**Conclusion:**

Our findings suggest that DUP is associated with temporal and occipitotemporal gray matter volume decrease in treatment naïve schizophrenia. The brain structural changes in untreated psychosis might contribute to poor treatment response and long-term prognosis in this patient population.

## Introduction

Brain gray matter volume abnormalities, primarily in the frontal and temporal lobes, have been consistently reported in first episode of psychosis [1], [2]. The exact mechanisms for brain abnormalities early in the course of illness are still poorly understood. In particular, it remains unclear whether such abnormalities are influenced by the duration of untreated psychosis (DUP). DUP is defined as the time between onset of psychosis and the start of hospitalization or adequate antipsychotic drug treatment [3], [4]. Prolonged DUP has been associated with poor clinical and social outcomes [5], [6]. It has been suggested that DUP may have neurotoxic effect on the brain [7]–[9].

Previous studies have examined the association between DUP and brain structural changes in schizophrenia [10]–[15]. In region of interest based analyses, a longer DUP was associated with decreased volumes in caudate nucleus [10], left planum temporal [11], and left superior temporal gyri [12].

The voxel-based morphometry (VBM) method, which is an automated whole-brain magnetic resonance image measurement technique, is capable of assessing anatomical differences in the entire brain, and avoids operational bias toward particular brain structures. Previous studies using VBM analysis reported that DUP correlated with decreased temporal gray matter volume in first episode psychosis [13], [14]. A recent study showed that DUP was associated with orbital-frontal gray matter volume decrease in patients with first episode psychosis [15]. But most of subjects in previous studies were on antipsychotic treatment at the time of imaging scan.

The present study was to use the VBM approach to examine the relationship between DUP and gray matter volume in antipsychotic medication treatment naïve schizophrenia.

## Methods

### Subjects

Antipsychotic medication treatment naive patients with schizophrenia were recruited from the Third Affiliated Hospital of Sun Yat-sen University, China. The diagnosis of schizophrenia was confirmed using the Structured Clinical Interview for DSM-IV Axis I Disorders–Clinician Version administered by clinicians (QL and YS)[16]. Patients had never received antipsychotic or other psychotropic medication treatment prior to the study. Healthy individuals with no psychiatric history from the local community, matched with the patient group by age, gender, handedness, and education level, were recruited as controls. All subjects were within the age range 18 to 45 years old, right-handed, of Han Chinese ethnicity, and had more than 9 years of formal education. The exclusion criteria included neurological disorders, traumatic brain injury, substance-related disorders, history of electroconvulsive therapy, family history of mental illness, mental retardation, and contraindications for MRI scanning.

The study was approved by the Institutional Review Board at the Third Affiliated Hospital of Sun Yat-sen University. A written informed consent was obtained from each subject, or his or her legal guardians.

### Clinical measures

The duration of untreated psychosis was evaluated using the Nottingham Onset Schedule (NOS) [17]. It was a short, guided interview and rating schedule to measure onset in psychosis. The NOS was completed based on medical records and the interview. The interview has explicit rules on how symptom onset and cessation should be rated, and if symptoms should be included. It also includes rules on choosing time frames when responses are unclear. The validity of the scale has been demonstrated in previous studies [18], [19].

The severity of psychopathology was assessed using the Positive and Negative Syndrome Scale (PANSS) [20].

### Imaging acquisition and processing

Brain MRI scans were acquired using a 1.5-T GE Signa Twinspeed MRI scanner (General Electric Medical System, Milwaukee, WI, USA) located in the Magnetic Resonance Center of the Third Affiliated Hospital of Sun Yat-sen University. The first sequence was a transverse spin-echo scan, which acquired both T2- and proton-density weighted images of the brain. High-resolution whole brain volumetric T1-weighted images were acquired sagittally with an inversion-recovery prepared 3-D spoiled gradient echo (SPGR) pulse sequence (TI = 650 ms, TE = 9.6 ms, flip angle = 15°, field-of-view, FOV = 240×240 mm^2^, slice thickness = 1.8 mm, matrix = 256×256, TR = 7.47 ms, slices = 301).

All structural data were processed using the VBM toolbox (VBM8) (http://dbm.neuro.uni-jena.de/vbm) in the Statistical Parametric Mapping 8 software package (http://www.fil.ion.ucl.ac.uk/spm). First, all T1-weighted anatomical images were normalized to T1 template in SPM and then segmented into gray matter(GM), white matter(WM), and cerebrospinal fluid images(CSF). After this preprocessing, segmented images were checked to ensure all images were unabridged. Imaging data were smoothed with an 8 mm full width at half-maximum (FWHM) Gaussian kernel before the GM images were entered into a statistical model.

### Statistical analysis

Clinical and demographic characteristics were compared among the groups using one-way analysis of variance (ANOVA) or chi-square test. A p value of 0.05 (two tailed) was used for statistical significance.

One-way analysis of covariance (ANCOVA) was used to compare VBM differences among the three groups controlling for gender, age and years of education, followed by post hoc t-tests for between group comparisons. The resulting statistical map obtained from ANCOVA test was corrected for multiple comparisons to a significant level of p< 0.01 by combining the individual voxel p <0.01 and cluster size >731 voxels using Monte Carlo simulations in the AFNI AlphaSim program.

To further examine the relationship between duration of untreated psychosis and the decreased gray volumes, we extracted the sum volumes of the clusters in the brain regions showed differences among 3 groups in VBM analysis. Multiple regression analysis in all patients was performed using SPM8 with gray matter volumes as dependent variable, DUP as a covariate of interest, and gender, age, years of education and PANSS total scores controlled as potential confounding variables.

## Results

### Demographic and clinical characteristics

A total of 61 patients and 32 healthy controls enrolled in the study. Four patients and 2 healthy controls withdrew from the study before any assessment procedures. Therefore 57 patients and 30 healthy controls completed the study.

According the previous study [15], patients were divided into two groups based on a DUP cutoff value of 26 weeks: the short DUP group (DUP≤26 weeks, n = 27) and the long DUP group (DUP>26 weeks, n = 30).

There were no significant differences in gender, age or years of education among the three groups (p's≥0.30). No significant differences were found between the two patient groups in the PANSS total or subscale scores (p's≥0.147; Table 1).

**Table 1:** Demographic and clinical characteristics of the study sample.

	Short-DUP(≤26 weeks)	Long-DUP(>26 weeks)	Healthy Controls	F/χ^2^	p
Age (years)	25.1(6.3)	25.7(6.7)	25.6 (6.7)	0.065	0.937
Gender (M/F)	16/11	16/14	16/14	0.264	0.876
Education (years)	11.3 (2.3)	12.1(2.9)	12.2(1.9)	0.996	0.374
DUP (weeks)	7.1 (1.4)	60.2 (11.0)	-	66.113	<0.001
PANSS positive scores	24.3 (4.9)	23.0 (6.3)	-	0.490	0.487
PANSS negative scores	13.8 (4.9)	16.0 (6.9)	-	2.165	0.147
PANSS total scores	73.7 (8.1)	75.3 (11.9)	-	1.642	0.205

Abbreviations: DUP = duration of untreated psychosis, PANSS =  the Positive and Negative Syndrome Scale, values are expressed as mean (SD).

### Gray matter volumetric measures

The VBM analysis showed significant differences among the three groups in gray matter volume in bilateral parahippocampus gyris, right superior temporal gyrus, left fusiform gyrus, left middle temporal gyrus, and right superior frontal gyrus (p's < 0.01).

**Figure 1:**
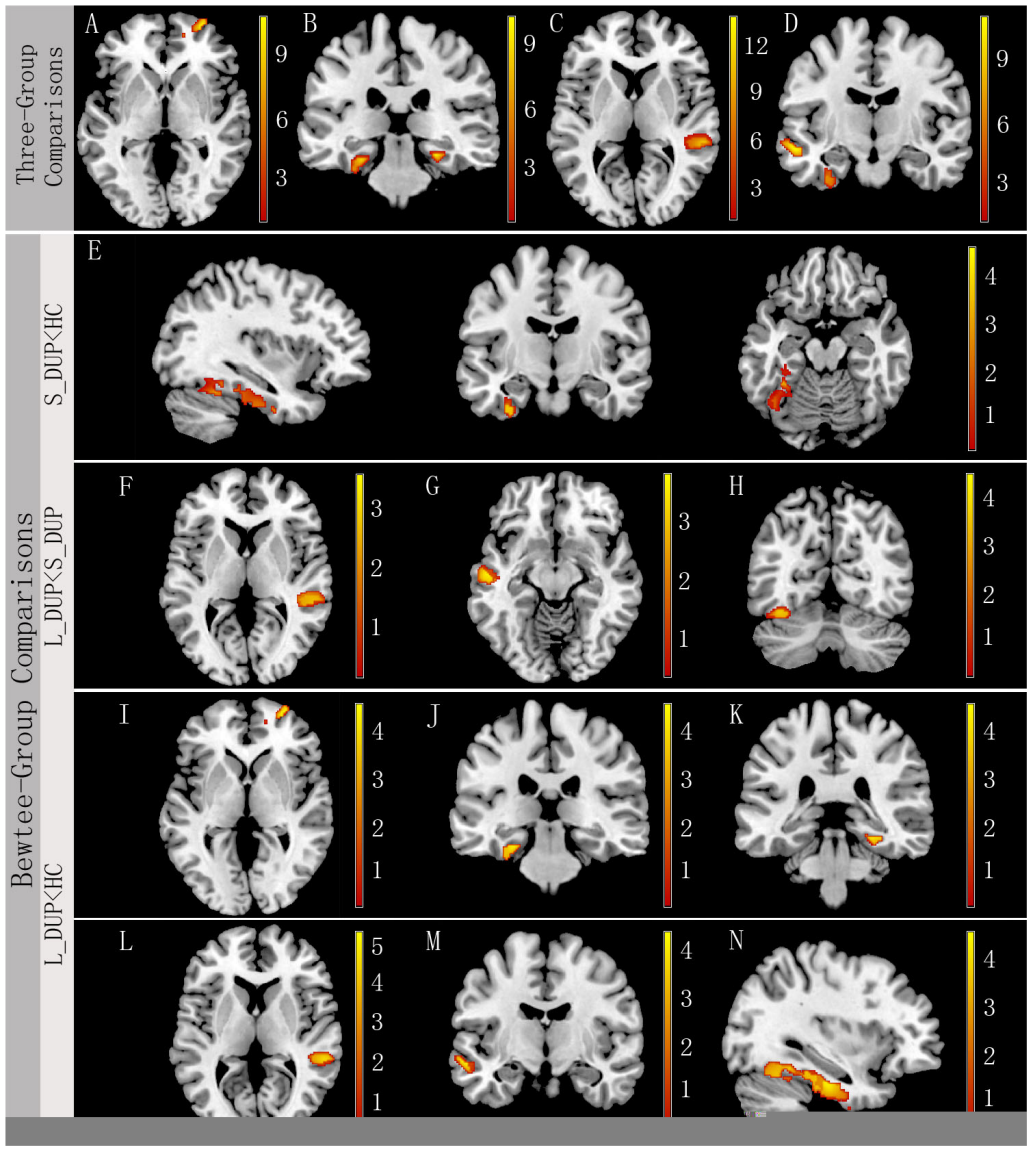
Brain anatomical regions that showed significant difference in gray matter volume among the three groups (right is right). A) right superior frontal gyrus, B) bilateral parahippocampus gyri, C) right superior temporal gyrus, and D) left fusiform and middle temporal gyri. Post-hoc analysis further suggested that the S-DUP group had significantly decreased gray matter volume in E) left fusiform gyrus compared with healthy control subjects, and significantly increased gray matter volume in F) right superior temporal gyrus, G) left middle temporal gyrus, and H) left fusiform compared with the L-DUP group. Compared with healthy control subjects, the L-DUP group had significantly decreased gray matter volume in I) right superior frontal gyrus, J) and K) bilateral parahippocampus gyrus, L) right superior temporal gyrus, M) left middle temporal gyrus, and N) left fusiform gyrus. Of note, statistical maps between L-DUP vs. Healthy control subjects were corrected for multiple comparisons using FDR (p<0.05), while those between L-DUP vs. S-DUP, and S-DUP vs. Healthy control subjects were corrected for multiple comparisons at the significant level of p<0.01 using AlphaSim Program (an extent threshold of p<0.01 with a minimum cluster size of 731 voxels).

Compared with healthy controls, long-DUP patients had significantly smaller gray matter volume in all these regions (p's <0.05, corrected). The gray matter volume was significantly smaller in right superior temporal gyrus, left fusiform gyrus, and left middle temporal gyrus in the long-DUP group compared with the short-DUP group(p's <0.01). Compared with healthy controls, the short-DUP group had a significantly smaller gray mater volume in left fusiform gyrus (p<0.01) (Fig. 1, Table 2).

**Table 2:** Comparison of gray matter volumes among the three groups.

				Comparison		
Anatomical region	MNI Coordinate(x, y, z)	Cluster size	F a	S-DUP<HC b	L-DUP<HC c	L-DUP <S-DUP b
L parahippocampal	−28.5, −22, −20	1278^d^	9.99		4.75	
R parahippocampal	27, −27, −15	1106	10.25		4.52	
L Fusiform	−37.5, −60, −16.5	1278^d^	11.67	4.33	4.57	4.28
L middle temporal	−54, −4.5, 16.5	756	7.12		4.25	3.89
R superior temporal	59, −31, 1	1323	13.90		5.26	3.78
R superior frontal	30, 62, 0	807	11.22		4.59	

Abbreviations: L = left, R = right, HC = Healthy Control subjects, S-DUP = short duration of untreated psychosis, L-DUP =  long duration of untreated psychosis.

^a^ All effects survived a voxel-wise statistical threshold using ANCOVA controlling for gender, age and years of education (p<0.01, AlphaSim corrected).

^b^ All effects survived a voxel-wise statistical threshold using post-hoc test (p<0.01, AlphaSim corrected).

^c^ All effects survived a voxel-wise statistical threshold using post-hoc test (p<0.05, FDR corrected).

^d^ These peaks belong to the same cluster.

### Relationship between DUP and gray matter volumetric measures

**Figure 2:**
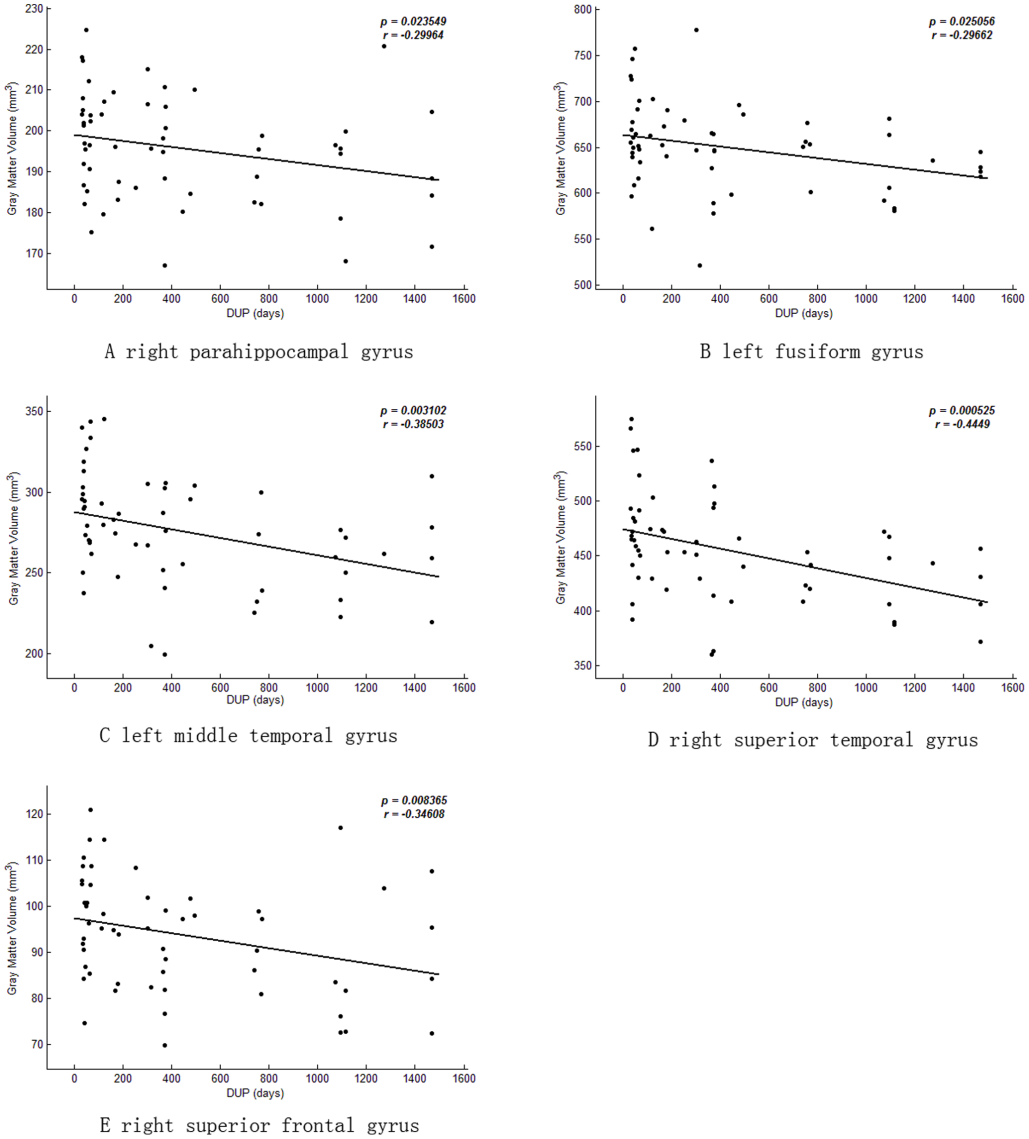
Association between duration of untreated psychosis and gray matter volume. Voxel-based multiple regression analysis was performed using SPM8 with gray matter volumes as dependent variable, DUP as a covariate of interest, and gender, age, years of education and PANSS total scores controlled as potential confounding variables.

Significant inverse relationships were found between DUP and right parahippocampal gyrus, right superior temporal gyrus, left fusiform gyrus, left middle temporal gyrus, and right superior frontal gyrus after controlling for gender, age, years of education, and PANSS total scores, indicating a longer DUP was associated with decreased gray matter volume in these regions (r≤−0.30, p's≤0.025; Fig. 2).

## Discussion

In this study, we examined the relationship between DUP and the brain gray matter volume in a sample of patients with antipsychotic medication treatment-naïve schizophrenia using voxel-based methods. Our results showed significantly smaller gray matter volume in right superior temporal gyrus, left fusiform gyrus, and left middle temporal gyrus in the long-DUP group compared with the short-DUP group. Our results are consistent with the findings from other studies [12]–[15], [21].

Several previous studies have shown that a prolonged DUP was associated with decreased superior temporal gyrus volume and left middle temporal gyrus volume [12], [15]. Superior temporal gyrus plays an important role, in thought and language processes, and is a major area of interest in schizophrenia research. One study has shown predominantly right-sided volumetric abnormalities in superior temporal gyrus in patients with early-onset schizophrenia [22].

Middle temporal gyrus is related to language and semantic memory processing, visual perception, and multimodal sensory integration. Previous studies have found middle temporal gyrus gray matter volume abnormalities in chronic and early-stage schizophrenia [23], [24]. A recent study has shown decreased left middle temporal gyrus volume in antipsychotic medication-naive, first-episode schizophrenia patients and their healthy unaffected siblings [25].

Lappin et al. [26] reported that a longer DUP was associated with poorer cognitive function in patients with schizophrenia. The temporal lobe has an integral role in language processing and semantic memory. The temporal gray matter volume abnormalities found in our study likely contributes to cognitive deficits in patients with untreated schizophrenia as reported in the literature. The fusiform gyrus, or occipitotemporal gyrus, is related to the processing and encoding of faces [27], which have been reported to be impaired in patients with schizophrenia [28]. Another prospective study has identified fusiform cortex reductions in individuals at high risk for psychosis [29]. A recent study found an inverse relationship between the volume of left fusiform gyrus and the duration of initial untreated period of first-episode psychosis [13]. The visual processing deficits observed in schizophrenia are associated with the abnormalities in this area [30]. Further, Chang et al. [31] found that a prolonged DUP was associated with more severe impairment in visual memory. Fusiform gyrus plays several essential roles in high-level visual processing and recognition. Our findings of fusiform abnormality are consistent with the functional impairment related to DUP as reported in previous studies [30], [31].

Increasing evidence suggests that the gray matter abnormalities associated with DUP may reflect the neurotoxic effect of untreated psychosis disease state [21]; gray matter volume continues to lose gradually throughout the course of illness if without intervention [32], [33]. As structural brain abnormalities are associated with poor treatment response and unfavorable long-term outcome [5], [6], early detection and intervention, therefore minimizing untreated time duration, become critically important in schizophrenia treatment.

In summary, our study examined the relationship between DUP and brain structure using an automated whole brain approach in a clinically well-characterized sample of treatment naive schizophrenia. We found that a prolonged DUP was associated with decreased temporal and occipitotemporal gray matter volumes. Our study has some limitations. The sample size was relative small. Since this was a cross-sectional study, a causal relationship between DUP and the brain abnormalities can’t be drawn. Future longitudinal studies are warranted to confirm our preliminary findings.
